# CD74 and HLA-DRA in Cervical Carcinogenesis: Potential Targets for Antitumour Therapy

**DOI:** 10.3390/medicina58020190

**Published:** 2022-01-26

**Authors:** Carol K. Balakrishnan, Gee Jun Tye, Shandra Devi Balasubramaniam, Gurjeet Kaur

**Affiliations:** 1 Institute for Research in Molecular Medicine, Universiti Sains Malaysia, Minden 11800, Pulau Pinang, Malaysia; josephcarol144@gmail.com (C.K.B.); geejun@usm.my (G.J.T.); shandrabala@gmail.com (S.D.B.); 2 Unit of Microbiology, Faculty of Medicine, AIMST University, Bedong 08100, Semeling, Malaysia

**Keywords:** CD74, HLA-DRA, cervical cancer, cervical intraepithelial neoplasia

## Abstract

*Background and Objectives*: Abnormal expressions of CD74 and human leukocyte antigen-DR alpha (HLA-DRA) have been reported in various cancers, though their roles in cervical cancer remain unclear. This study aimed to evaluate the gene and protein expressions of CD74 and HLA-DRA in the progression from normal cervix to precancerous cervical intraepithelial neoplasia (CIN) and finally to squamous cell carcinoma (SCC). *Materials and Methods*: The gene expression profiles of CD74 and HLA-DRA were determined in formalin-fixed paraffin-embedded tissues, with three samples each from normal cervixes, human papillomavirus type 16/18-positive, low-grade CIN (LGCIN), high-grade CIN (HGCIN), and squamous cell carcinoma (SCC) using Human Transcriptome Array 2.0. Immunohistochemical expression of the proteins was semi-quantitatively assessed in another cohort of tissue microarray samples comprising 7 normal cervix cases, 10 LGCIN, 10 HGCIN, and 95 SCC. *Results*: The transcriptomics profile and proteins’ expression demonstrated similar trends of upregulation of CD74 and HLA-DRA from normal cervix to CIN and highest in SCC. There was a significant difference in both proteins’ expression between the histological groups (*p* = 0.0001). CD74 and HLA-DRA expressions were significantly associated with CIN grade (*p* = 0.001 and *p* = 0.030, respectively) but not with the subjects’ age or SCC stage. Further analysis revealed a positive correlation between CD74 and HLA-DRA proteins. *Conclusions*: CD74 appears to promote cervical carcinogenesis via oncogenic signalling mechanisms and may serve as a potential antitumour target. Additionally, the upregulation of HLA-DRA, often associated with stronger immunogenicity, could be a promising biomarker for developing immunotherapies.

## 1. Introduction

Cervical cancer is the fourth most common cancer and the fourth leading cause of cancer death in women worldwide [1]. The analysis of cancer biomarkers that can be differentially expressed in the presence of this severe disease is an enormous challenge for researchers. Despite routine cytological screening programmes and the availability of multiple modalities of treatment, late-stage disease is associated with a poor outcome [2,3]. There is an urgent need to develop novel molecular therapeutic targets for the treatment of precancerous lesions and cervical cancer to reduce its mortality. Human papillomavirus (HPV), the causative factor in 90% of cervical cancers [4], integrates into host cervical cells and causes molecular alterations that result in cell proliferation and the impairment of apoptosis and DNA repair mechanisms. Hence, the progression of HPV-infected cells into cervical intraepithelial neoplasia (CIN) and finally into invasive cancer [5].

Major histocompatibility complex (MHC) class II proteins specialise in the presentation of peptide antigens to CD4+ T cells [6]. Though MHC-II molecules are predominantly expressed by professional antigen-presenting cells, such as dendritic cells, B cells, and macrophages, recent evidence shows that a variety of tumours also express them, including breast cancer [7,8], colorectal cancer [9,10], ovarian cancer [11], and melanoma [12]. CD74 is the membrane form of the MHC class II invariant chain (Ii). When MHC class II–CD74 complexes are formed, they are rapidly internalised to the endoplasmic reticulum for reloading with peptides to be recycled to the cell surface, triggering the adaptive immune response [13,14]. Recently, CD74 has been described as having an oncogenic role in promoting cell proliferation and preventing cell death via a macrophage migratory inhibitory factor (MIF)-dependent manner [15], as proven in breast cancer [16], renal cell carcinoma [17], colorectal cancer [18], and non-small-cell lung carcinoma [19].

HLA-DR is involved in the suppression of tumour growth, whereby it presents tumour-associated antigens (TAA) that are recognised by CD4+ T cells, which then produce cytokines, such as interleukins and interferon-γ (IFN-γ), to inhibit tumour growth [20,21]. Conflicting results have been reported on the expression of HLA-DRA in various cancers. Overexpression of HLA-DRA was demonstrated in colorectal cancer [22], hepatocellular cancer [23], and ovarian cancer [24], while it was downregulated in breast cancer [8]. HLA-DRA has also been shown to act as a prognostic marker for clinical outcomes [13,25].

The roles of CD74 and HLA-DRA in cervical cancer development are relatively unknown. We aimed to evaluate the gene and protein expressions of these molecules in the progression from normal cervix to precancerous cervical intraepithelial neoplasia (CIN) and finally to squamous cell carcinomas (SCC), with the intent of exploring their potential use as therapeutic targets. To the best of our knowledge, this is the first report on the transcriptomic and protein expressions of these targets in the development of cervical cancer.

## 2. Materials and Methods

### 2.1. Tissue Samples, RNA Extraction, Transcriptome Array

**Figure 1:**
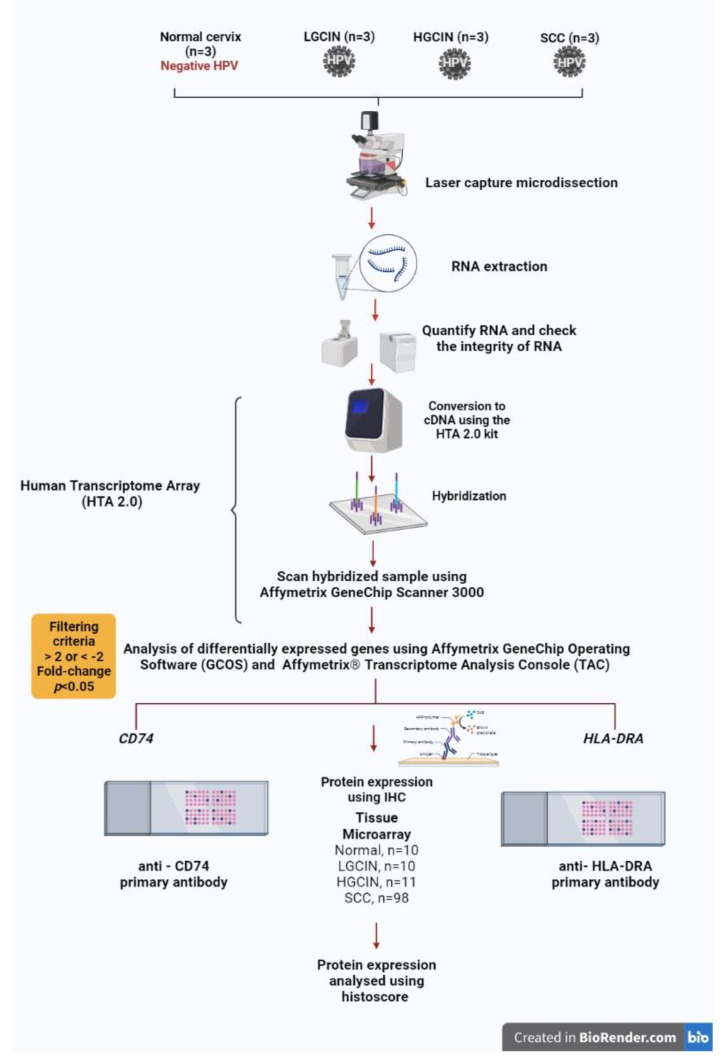
Methodology workflow of the study.

Three cases each of human papillomavirus (HPV) 16/18-positive, low-grade cervical intraepithelial neoplasia (LGCIN), high-grade cervical intraepithelial neoplasia (HGCIN), squamous cell carcinoma (SCC), and HPV-negative normal cervixes were included in the study after approval by the Human Research Ethics Committee of Universiti Sains Malaysia, and in accordance with the Code of Ethics by the World Medical Association. Formalin-fixed, paraffin-embedded tissue blocks were retrieved from the pathology department archives. HPV 16/18-positive samples were identified by immunohistochemistry with p16INK4a and HPV16 E6 + HPV18 E6 antibodies and real-time PCR. The areas of interest within the squamous epithelium were dissected using a ZEISS PALM Microbeam Laser Microdissection System (Carl Zeiss) from two 10µm-thick tissue sections. Then, total RNA was extracted from the tissues with RNeasy FFPE extraction kit (Qiagen, Hilden, Germany). GeneChip Human Transcriptome Array 2.0 (HTA 2.0) (Affymetrix, Santa Clara, CA, USA) was used to profile the gene signatures of the four histological groups. Sensation plus FFPE WT kit (Affymetrix, Santa Clara, CA, USA) was used according to the manufacturer’s protocol. Upon hybridisation, the chip was scanned using Affymetrix GeneChip Scanner 3000. The data were analysed with Affymetrix GeneChip Operating Software (GCOS), which contains qualitative and quantitative analysis for every probe set. Affymetrix Transcriptome Analysis Console (TAC) software was used to determine CD74 and HLA-DRA transcriptomic profiles in each histological group compared to a normal cervix. The methodology workflow is illustrated in Figure 1.

### 2.2. Immunohistochemical Expression of CD74 and HLA-DRA Proteins in Tissue Microarrays

Immunohistochemistry (IHC) for each protein was performed on 2 tissue microarrays (TMA) (BB10011 and CR1101, Biomax, Rockville, MD, USA) comprising 10 cases of normal cervixes, 10 LGCIN, 11 HGCIN, and 98 squamous carcinoma cases. The TMAs had accompanying data on pathology diagnosis, CIN grade (Bethesda Classification System), and International Federation of Gynecology and Obstetrics (FIGO) staging for cervical cancer cases.

IHC was performed using the horseradish peroxidase polymer method from EnVision™ FLEX/HRP (Dako, Via Real Carpinteria, CA, USA). After the TMA slides were dewaxed and rehydrated, they were immersed in EnVision™ FLEX target retrieval solution (50× Tris/EDTA buffer, pH 9) and in a microwave oven (Panasonic) under a low setting for 20 min. Subsequently, the slides were incubated with a few drops of EnVision™ FLEX Peroxidase solution for 5 min, followed by a rinse. Rabbit polyclonal anti-CD74 antibody (1:500 dilution; cat. no. HPA010592, Sigma-Aldrich, St. Louis, MO, USA) or rabbit polyclonal anti-HLA-DRA antibody (1:1000 dilution; cat. no. HPA053176, Sigma-Aldrich, USA) was applied and incubated overnight at 4 °C. After rinsing, EnVision™ FLEX/HRP secondary antibody was added and incubated for 20 min followed by EnVision™ FLEX substrate working solution (EnVision™ FLEX substrate buffer mixed with EnVision™ FLEX DAB + chromogen) for 5 min. Slides were counterstained with Mayer’s haematoxylin for 5 min. The slides were rinsed thrice with EnVision™ FLEX Wash Buffer for 5 min each between the incubation steps above. Finally, the slides were dried, mounted, and viewed under a light microscope before they were scanned with a Pannoramic Digital Slide Scanner (3DHISTECH Ltd., Budapest, Öv u. 3., Hungary). The digital slides from whole slide imaging were analysed using Case Viewer software. Brown staining signified positive protein expression. The histoscore was calculated by multiplying the percentage of the positivity score and staining intensity score, as shown in Table 1. Tonsil tissue was used as a positive control, where brown staining was observed in lymphocytes of lymphoid follicle germinal centres. Primary antibodies were omitted in the negative control.

### 2.3. Statistical Analysis

Transcriptomic analysis in HTA 2.0 was performed by comparing the gene expression in each histological group to normal cervixes, using a one-way ANOVA test provided by the array software, with a significance set at a *p* value < 0.05. The association between CD74 and HLA-DRA histoscore and histological groups as well as clinicopathological parameters was analysed by the Pearson chi-square test with the significance set at a *p* value < 0.05, using the IBM SPSS v26.0 software package for Windows. Further analysis of the correlation between CD74 and HLA-DRA protein expression was calculated using Spearman’s rank correlation with the significance at a *p* value < 0.01.

## 3. Results

### 3.1. Transcriptomics Profile

**Figure 2:**
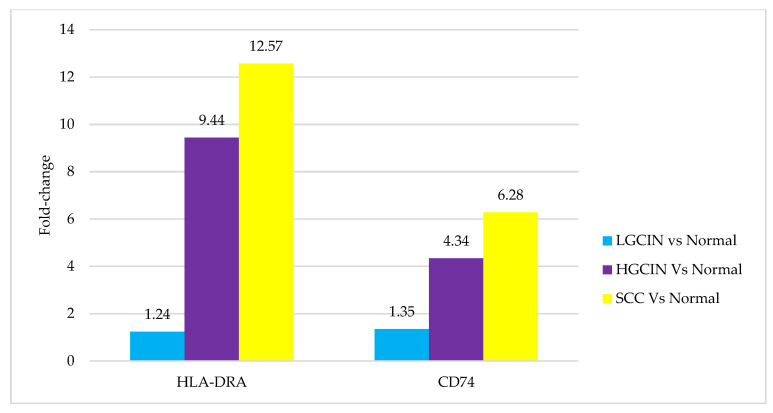
Transcriptomic profiles of HLA-DRA and CD74 in LGCIN, HGCIN, and SCC obtained from Human Transcriptome Array. Bars represent the fold change difference in each histological group compared to the normal cervix. The fold changes were not significant by one-way ANOVA where *p* > 0.05.

The transcriptomic profile of CD74 and HLA-DRA genes was compared between each histological group (LGCIN, HGCIN, and SCC) and the normal cervixes. The differences in fold change are shown in Figure 2. The results demonstrate an increasing trend in upregulation in both genes, from LGCIN to HGCIN and highest in SCC. The difference in fold change was minimal in LGCIN compared to normal cervixes (1.24 for HLA-DRA and 1.35 for CD74). As the disease progressed, the genes were increasingly upregulated. The HLA-DRA gene showed a 9.44-fold change in HGCIN and a 12.57-fold change in SCC, while the CD74 gene revealed a 4.34-fold change in HGCIN and a 6.28-fold change in SCC, compared to the normal cervixes. However, it should be noted that the fold change differences were not statistically significant.

### 3.2. Immunohistochemistry

**Figure 3:**
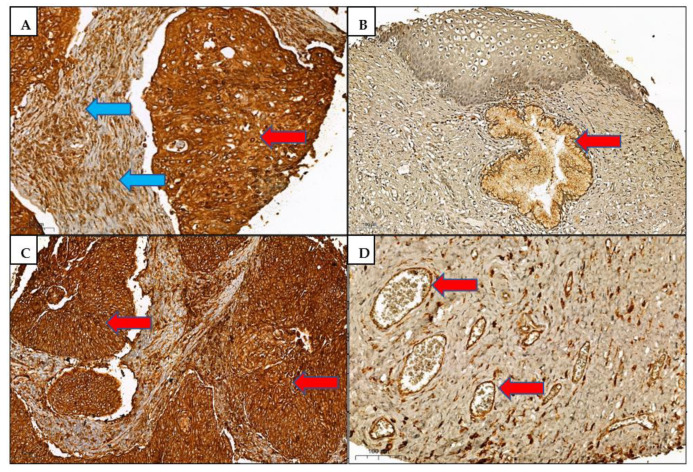
Immunohistochemical expression patterns in tissue microarrays. (**A**) CD74 high histoscore in cytoplasm/membrane of SCC cells (red arrow) and stromal lymphocytes (blue arrows). (**B**) Absent CD74 expression in normal squamous cells and low expression in normal endocervical cells (red arrow). (**C**) HLA-DRA high histoscore in cytoplasm/membrane of SCC cells (red arrows). (**D**) HLA-DRA positivity in endothelial cells of small blood vessels (red arrows). Scale bar: 100 µm.

The CD74 and HLA-DRA proteins’ expressions were scored on 122 cases comprising 7 normal cervixes, 10 LGCIN, 10 HGCIN, and 95 SCC cases. Seven tissue cores were excluded due to the absence of squamous cells. We noted that both proteins were localised in the cytoplasm and membrane of squamous cells. Stromal inflammatory cells and endothelial cells of blood vessels were positively stained. The association between protein histoscore and histological groups as well as clinicopathological parameters are tabulated in Table 2 and Table 3. Both CD74 and HLA-DRA immunohistochemical expressions were significantly different between the histological groups (*p* = 0.0001) and in SCC compared to normal cervixes (*p* = 0.0001). Additionally, the CD74 expression was significantly different in HGCIN compared to the normal cervixes (*p* = 0.006), and HLA-DRA was differently expressed in LGCIN compared to the normal cervixes (*p* = 0.001). Both proteins showed significant association with CIN grade, but not with the subject’s age or SCC stage. The correlation coefficient (rs) of CD74 and HLA-DRA expression was 0.76, *p* = 0.0001, reflecting a positive correlation between the two proteins. Representative immunohistochemical staining patterns are shown in Figure 3.

## 4. Discussion

The progression of HPV-infected cervical cells into a distinctive precancerous stage (CIN) and finally into SCC offers a unique advantage to study the roles of CD74 and HLA-DRA molecules in cervical carcinogenesis. Numerous reports suggest these molecules are involved in the pathogenesis of various cancers. This is the first study elucidating the possible roles of these target genes and proteins in cervical cancer development. CD74 is believed to exert an oncogenic effect. The present study demonstrated an increasing trend in CD74 gene and protein expression, being lowest in normal cervixes, higher in CIN, and highest in the SCC group (Figure 2 and Table 1). These findings are in concordance with another report of higher CD74 protein expression in CIN lesions compared to normal cervixes and higher in SCC than in CIN [26]. An increased expression of CD74 in human colorectal adenomas was found to be related to an ascending grade of epithelial cell dysplasia, corresponding to the CIN stage in our study [27]. Furthermore, CD74 immunohistochemical expression increased in higher-grade and late-stage metastatic invasive cancers compared to less aggressive and lower-grade cancers, as reported in thymic epithelial neoplasms [28], colorectal, gastric, and pancreatic cancers [29], and urothelial carcinoma of the bladder [17]. The presence of CD74 protein on tumour cells membranes and cytoplasm in our study suggests an interaction with macrophage migratory inhibitory factor (MIF) and internalisation of CD74, in agreement with other studies [16,17,28].

In our study, CD74 expression was not associated with the patient’s age (*p* = 0.083) or SCC stage (*p* = 0.790) (Table 3). There are no published reports in the literature on these traits in cervical cancer. However, in urothelial carcinomas of the bladder, there was a correlation with older subjects and an advanced stage of disease [17]. Further analysis of a larger cohort is necessary to gain an insight into the possible predictive role of CD74 in cervical cancer in different patient age groups and cancer stages.

The upregulation of CD74 in many types of cancers suggests its role in facilitating tumour progression and metastasis. Upon stimulation by the migration inhibitory factor (MIF), CD74 coupled with CD44 can induce phosphorylation and activation of Src and ERK1/2, as well as p53 dephosphorylation, eventually promoting cell proliferation and inhibiting apoptosis [14,30]. Persistent overexpression of CD74 on the cell surface could impair MHC class II antigen presentation by tumour cells, thereby contributing to immune escape and promoting tumour metastasis [15]. It has also been demonstrated that CD74’s cytoplasmic domain binds chromatin and regulates the transcription and expression of genes involved in immune regulation, cell survival, and hematopoietic cancers [31]. CD74 may have a potential role in targeted cancer therapy, using monoclonal antibodies or small molecules to block CD74 function [15].

In the present study, HLA-DRA gene expression was lowest in LGCIN and highest in SCC, compared to normal cervixes (Figure 1), correlating with the immunohistochemistry results, with absent expression in 80% of LGCIN cases and highly expressed in 88.4% of SCC cases (Table 2). The findings suggest immune escape, which would promote the proliferation of HPV-infected cells in the LGCIN stage. As the disease progresses to HGCIN and SCC, increased synthesis of HLA-DRA protein recruits immune support via CD4+ T cells, leading to more favourable CD8+ T-cells responses and thus preventing tumour invasion. This finding parallels a previous study using the same sample cohort that revealed deregulation of the immune-related genes, including MHC II and immunoglobulin heavy chain genes in the progression from LGCIN to HGCIN and SCC [32].

The precise role of HLA class II in cancer is poorly understood, reflected by conflicting reports on its protein expression in various cancers. Strong HLA-DR expression has been demonstrated in colorectal cancer tissues and was related to a better prognosis [22], similarly in ovarian cancer [33], and consistent with our study. An upregulation of HLA-DRA expression is associated with antitumour immune response, whereby it functions in presenting tumour-associated antigens (TAA) on cancer cells that trigger CD4+ Th1 cells and NK cells, with the production of IFN-γ, an antitumour cytokine. A causative relationship has been found between MHC-II and/or HLA-DRA and increased tumour-infiltrating lymphocytes, associated with more favourable outcomes in cancer patients [13,25,34]. However, it is still unclear whether this type of immune response determines the rate of tumour progression or an impression of ongoing elimination or equilibrium phases of cancer immunoediting [35]. In our study, HLA-DRA expression was not associated with the SCC stage (*p* = 0.753) (Table 3). Additionally, we could not evaluate the amount of tumour-infiltrating lymphocytes in the small tissue cores. The therapeutic implications of tumour-specific MHC-II (tsMHC-II) are promising, wherein their upregulation correlates with a better response to an anti-PD-1 monoclonal antibody in Hodgkin lymphoma and melanoma [12,36], and therefore offering opportunities as a biomarker in the development of immunotherapies [13,25]. Additional studies are required to evaluate the role of MHC-II and PD-1/PD-L1 to predict the treatment response in cervical cancer.

In contrast, downregulation of HLA-DR is associated with an unfavourable outcome and aggressiveness in breast cancer [8], diffuse large B cell lymphoma [37], and small-cell lung carcinoma (SCLC) compared to non-SCLC [38]. A low expression observed in hepatocellular carcinoma was linked to early intrahepatic recurrence [23]. Repression of tumour-specific MHC-II molecules in cancers, often by hypermethylation or hypoacetylation of promoters of HLA genes and/or class II transactivator (CIITA), causes a reduction in T helper 1 (Th 1) cytokines such as IFN-γ and TNFα, which inhibit the activation of CD8+ cytotoxic T cells, eventually leading to a loss of tumour immunosurveillance [13,25,37,39].

The positive correlation between the CD74 and HLA-DRA expression in our study was also found in another report on invasive thymomas [28]. This suggests that overexpressed CD74 did not affect the antigen presentation machinery of HLA-DRA. In contradiction, CD74 or the invariant chain (Ii) expression was negatively correlated with HLA-DR expression in gastric cancer [40] and breast carcinoma [41]. This may be due to superinduction of Ii affecting tumour antigen presentation by MHC-II molecules, contributing to immune evasion and tumour metastasis [42].

Notwithstanding the small number of clinical samples used in transcriptomics analysis, the method used is sensitive, and the results clearly indicated an upward trend of CD74 and HLA-DRA gene expression from normal cervixes to CIN and SCC. The validation by protein expression analysis in a large adequate sample size supported these findings.

## 5. Conclusions

**Figure 4:**
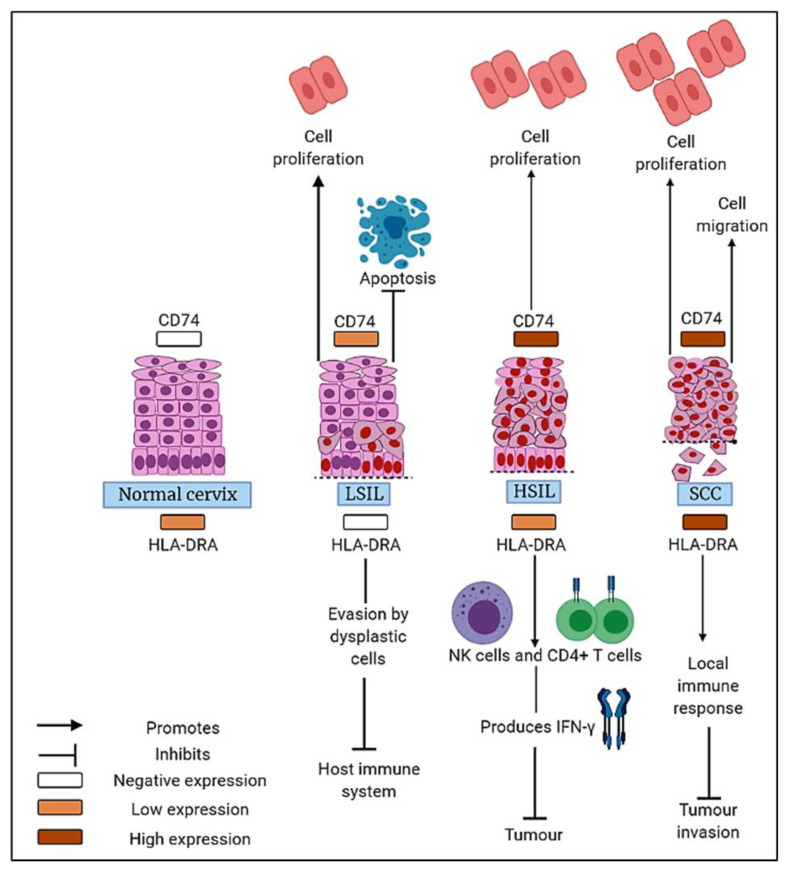
Summary of results illustrating CD74 and HLA-DRA expression patterns and their potential roles in the development of cervical cancer.

Our findings suggest that CD74 protein promotes cervical carcinogenesis via oncogenic signalling mechanisms and could be potentially useful for antitumour therapy. CD74 may facilitate HLA-DR internalisation to trigger stronger immunogenicity against tumours. HLA-DRA may aid as a prognostic biomarker and play a possible role in immunotherapy. Our hypotheses of the possible effects of CD74 and HLA-DRA are illustrated in Figure 4. Future trends incorporating high throughput analysis of gene and protein expression in tandem with patients’ clinical outcomes will lead to the discovery of potential targets for immunotherapy and targeted therapy directed towards precision medicine.

## Figures and Tables

**Table 1:** Semi-quantitative scoring for immunohistochemical expression of CD74 and HLA-DRA proteins.

Percentage of Positive Cells	Score	Staining Intensity	Score
<1% positive cells	0	No staining	0
1–25% positive cells	1	Weak staining	1
26–50% positive cells	2	Moderate staining	2
51–75% positive cells	3	Strong staining	3
≥75% positive cells	4		
Final score = percentage of positive cells score × staining intensity score. Final histoscore: 0 = negative. 1 to 3/12 = low expression. ≥4/12 = high expression.			

**Table 2:** Comparison of CD74 and HLA-DRA histoscores between the histological groups.

	Histoscore					
Histological Group	Negative *n* (%)	Low *n* (%)	High *n* (%)	Total *n* (%)	Comparison between All Histological Groups *p* Value	Histological Group vs. Normal Cervix *p* Value
CD74 protein						
Normal cervix	4 (57.1)	2 (28.6)	1 (14.3)	7 (100)	0.0001	
LGCIN HGCIN	4 (40.0) 1 (10.0)	5 (50.0) 0 (0.0)	1 (10.0) 9 (90.0)	10 (100) 10 (100)		0.798 0.006
SCC	5 (5.3)	7 (7.4)	83 (87.4)	95 (100)		0.0001
Total	14 (11.5)	14 (11.5)	94 (77.0)	122 (100)		
HLA-DRA protein						
Normal cervix	0 (0.0)	6 (85.7)	1 (14.3)	7 (100)	0.0001	
LGCIN HGCIN	8 (80.0) 2 (20.0)	1 (10.0) 5 (50.0)	1 (10.0) 3 (30.0)	10 (100) 10 (100)		0.001 0.464
SCC	6 (6.3)	5 (5.3)	84 (88.4)	95 (100)		0.0001
Total	16 (13.1)	17 (13.9)	89 (73.0)	122 (100)		

Chi-square test, statistical significance set at *p* value < 0.05.

**Table 3:** The association between CD74 and HLA-DRA proteins expression with demographic and clinicopathological parameters.

Parameters	CD74 Histoscore			Total *n* (%)	*p* Value	HLA-DRA Histoscore			Total *n* (%)	*p* Value
	Negative *n* (%)	Low *n* (%)	High *n* (%)			Negative *n* (%)	Low *n* (%)	High *n* (%)		
Age (years) <50	4 (5.8)	8 (11.6)	57 (82.6)	69 (100)	0.083	10 (14.5)	10 (14.5)	49 (71.0)	69 (100)	0.838
≥50	10 (18.9)	4 (7.5)	39 (73.6)	53 (100)		6 (11.3)	7 (13.2)	40 (75.5)	53 (100)	
CIN grade LGCIN	4 (40.0)	5 (50.0)	1 (10.0)	10 (100)	0.001	8 (80.0)	1 (10.0)	1 (10.0)	10 (100)	0.030
HGCIN	1 (10.0)	0 (0.0)	9 (90.0)	10 (100)		2 (20.0)	5 (50.0)	3 (30.0)	10 (100)	
SCC stage Stage I	0 (0.0)	2 (6.7)	28 (93.3)	30 (100)	0.790	1 (3.3)	0 (0.0)	29 (96.7)	30 (100)	0.753
Stage IA	0 (0.0)	0 (0.0)	1 (100.0)	1 (100)		0 (0.0)	0 (0.0)	1 (100)	1 (100)	
Stage IB	3 (15.0)	2 (10.0)	15 (75.0)	20 (100)		2 (10.0)	2 (10.0)	16 (80.0)	20 (100)	
Stage IC	0 (0.0)	0 (0.0)	1 (100.0)	1 (100)		0 (0.0)	0 (0.0)	1 (100)	1 (100)	
Stage II	0 (0.0)	1 (16.7)	5 (83.3)	6 (100)		0 (0.0)	0 (0.0)	6 (100)	6 (100)	
Stage IIA	1 (5.3)	1 (5.3)	17 (89.5)	19 (100)		1 (5.3)	1 (5.3)	17 (89.5)	19 (100)	
Stage IIB	1 (8.3)	1 (8.3)	10 (83.3)	12 (100)		2 (16.7)	1 (8.3)	9 (75.0)	12 (100)	
Stage III	0 (0.0)	0 (0.0)	1 (100.0)	1 (100)		1 (3.3)	0 (0.0)	29 (96.7)	30 (100)	
Stage IIIB	0 (0.0)	0 (0.0)	4 (100.0)	4 (100)		0 (0.0)	0 (0.0)	1 (100)	1 (100)	

Chi-square test, statistical significance set at *p* value < 0.05.

## Data Availability

The datasets generated during and/or analysed during the current study are available from the corresponding author on reasonable request.
